# Predictive stochastic modeling of mechanically alloyed particle size and shape

**DOI:** 10.1039/d5ra03243a

**Published:** 2025-06-18

**Authors:** Anand Prakash Dwivedi, Emad Iranmanesh, Katerina Sofokleous, Vassilis Drakonakis, Amin Hamed Mashhadzadeh, Maryam Zarghami Dehaghani, Boris Golman, Christos Spitas, Charalabos C. Doumanidis

**Affiliations:** a Mechanical Engineering and Robotics, Guangdong Technion Israel Institute of Technology Shantou China anand.dwivedi@gtiit.edu.cn; b School of Electronic and Computer Engineering, Peking University Shenzhen Graduate School Shenzhen China iranmanesh.emad@yahoo.com; c AMDM Composites Nicosia Cyprus katerina@amdmcomposites.com vassilis@amdmcomposites.com; d Institute for Materials and Processes, School of Engineering, University of Edinburgh Edinburgh Scotland UK maryam.zarghamid@gmail.com; e Department of Chemical and Materials Engineering, Nazarbayev University Kazakhstan boris.golman@nu.edu.kz; f Department of Mechanical Materials and Manufacturing Engineering, University of Nottingham Ningbo China christos.spitas@nottingham.edu.cn; g Senior Technical Advisor, AMDM Composites Nicosia Cyprus hdoumani@gmail.com; h Department of Mechanical Engineering, Indian Institute of Technology Kanpur Kanpur India; i Department of Mechanical Engineering, Nazarbayev University Kazakhstan amin.hamed.m@gmail.com

## Abstract

Mechanical alloying of bimetallic materials by ball milling produces particulate products where, aside from internal structure, the size and shape of particles is of importance for various applications. This article introduces real-time modeling tools for the particle species demographics of size and aspect ratio, their dynamic evolution and dependence on processing conditions. Its highlight is a simple, analytical stochastic model of external particle features based on statistical formulations of impact energetics, friction and plastic deformation effects, as well as bonding and fracture transformations of the particles during the process. The model is calibrated and validated experimentally by measurements on laboratory micrographs and literature data in low- and high-energy ball milling of Al–Ni powders at different molar ratios. Its size and shape predictions offer insights to population growth of particles through mechanical alloying phenomena for material design and optimization and process observation for real-time feedback control.

## Abstract 2

Ball milling bimetallic materials creates tailored particles—size, shape, and nanostructure define performance for catalysis, energy, and beyond.

## Graphical abstract

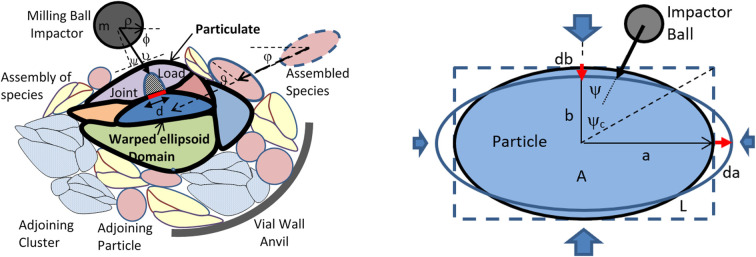

## 1. Introduction

Mechanical alloying of metallic and composite particulate materials by ball milling (BM) has been a popular synthesis and batch fabrication method over the past few decades, because of its simplicity, flexibility and affordability.^1–3^ A variety of magnetic intermetallic nanostructures,^4–6^ reactive bimetallic and thermite nanoheaters^7–9^*etc.* have been successfully fabricated by BM and tested in hard and super-paramagnetic, self-heated micro-soldering and other applications.^10^ Emphasis has been given to the study of internal structure and composition of these mechanically alloyed particles, as they directly relate to their structural, magnetic and thermal properties and function.^11,12^ Such investigations have shown that structural evolution of globular particle agglomerates in the original metallic powders into micro/nanoscale lamellar networks in the final composites during BM plays a key role in their diffusion, heat, electric and magnetic field transport during operation, therefore allowing for new engineered and custom-designed functionalities.^10^

Comparatively less attention has been paid in the literature to the size and shape distributions of such intermetallic particulates and their evolution during mechanical alloying, *e.g.* of high-energy BM Al–Ni powders.^13^ Spinel Fe_3_O_4_ nanoparticle size profiles produced by low-energy BM have been shown to follow log-normal distributions,^4^ and ternary Al–V–Cu, Ni, Mn mechanical alloyed nanoparticles imaged by TEM were synthesized.^5^ The time evolution of size and composition of Sm–Co particles prepared by BM was also assessed,^6^ and that of Ni–Ta binary particulates was investigated.^14^ Reduction of particle size during planetary BM was reported,^15^ while for ball grinding and stirred BM the diminishing mean size and increasing standard deviation of log-Gaussian particle distributions with processing time and power was also studied experimentally.^16^ The shape evolution of ellipsoid particles and their planarization or spheronization upon random or flow-directed ball impacts was investigated by simulation.^17^ Last, qualitative insights to deformation, micro-welding and fracturing mechanisms of BM particles leading to their Gaussian-like distributions and their dynamics were illustrated.^2^

However, the approach in the previous bibliography has been predominantly either descriptive, *i.e.* towards empirical models by fitting of laboratory data, or theoretical, *i.e.* towards computational deterministic simulations of particle deformation. This leaves an outstanding need for predictive models of particulate size and shape distributions from their BM processing conditions, calibrated and validated through experimental results, and based on stochastic numerical formulations. Such predictive modeling is enabled by recent development of a dynamic computational simulation of the internal particulate structure evolution during BM.^18^ This real-time computationally efficient model simulates the mechanics of powder and cluster particle impact, contact, friction, deformation, assembly, integration and fracturing upon random collisions with the vial walls and milling balls of known energetics.^19^

**Fig. 1:**
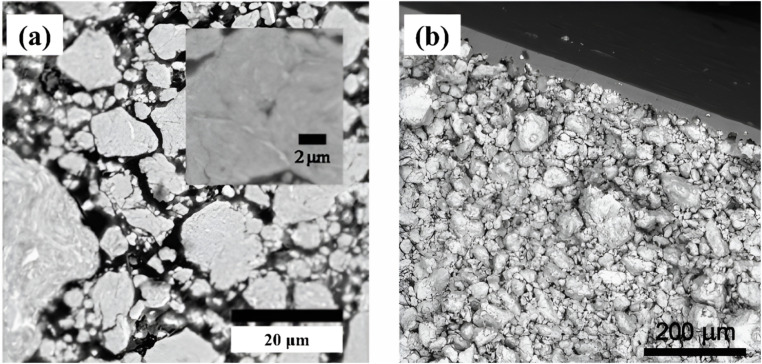
SEM micrographs of Al–Ni (1 : 1) composite particulates by BM (300 rpm): (a) loose particulate external and internal structure (after 13 hours of BM)^10^ (b) compacted into 800 μm-thick foils sandwiched with Al overlayers (9 hours of BM).^12^

By contrast, the novelty of this present work is in establishing a parsimonious analytical, stochastic predictive, true real-time model to estimate the external particle size and shape of bimetallic BM particulates, which to the authors' knowledge is unavailable in the literature. This provides a valuable tool for design and optimization of compacted pellet products from BM particles, such as loose Al–Ni particulates (Fig. 1a), *e.g.* into compressed foils clad with external Al layers (Fig. 1b). Upon local ignition of such cold-bonded compacts, the self-propagating exothermic reaction of the BM particulates^10,12^ depends on conductive heat and diffusive mass transfer across their joint boundary surfaces. The local area and direction of these interfaces, as well as their thermal resistance, is determined by the original size and shape of the particle components. In addition, the model also establishes a novel in-process observer of the experimentally inaccessible BM process in real time, as the basis for much-needed adaptive process control schemes, to deliver the specified size and shape of the particulate product while still processed.

Therefore, this article addresses the stochastic formulation, experimental calibration and laboratory testing of such predictive models of BM particle size and shape, based on the previous numerical simulation and empirical data of the literature. Section 2 describes the laboratory BM conditions for the Al–Ni mechanical alloying experiments, as well as the fundamental underpinnings of the real-time structural model, with emphasis on the probabilistic collision, directional assembly and demographic classification of random particle components. Section 3 establishes a stochastic formulation for population growth of particulate species with various size and size distributions through friction, plastic deformation, bonding and fracture during BM. Section 4 describes model calibration and validation through literature data and laboratory results, and obtains insights from simulation results. Finally, Section 5 summarizes conclusions and further steps towards utilization of the outcomes towards property design of particulate products and BM process control.

## 2. Experimental and computational methods

### 2.1 Experimental setup

The well-studied in the literature bimetallic Al–Ni system,^11,13^ known for formation of ignitable, exothermically reactive particulates with internal lamellar nanostructures^10,12^ (Fig. 1), was selected for the experiments. Al (99.97% purity) and Ni (99.8% purity) powders (Alfa Aesar)^24–26^ with initial narrow size distribution at −325 mesh (40 + 4 μm) were used at molar ratios of 1 : 1 or 1 : 3, and total loading mass of 32 g.^27–30^ The Brunauer Emmett Teller (BET) nitrogen adsorption/desorption isotherm-based specific surface area of these mixtures was determined to 112–138 m^2^ g^−1^.^31–35^ Their texture analysis by selected area electron diffraction (SAED) is also available in the literature.^36–39^ Dry planetary ball milling (Fritsch Pulverisette) was performed in continuous low-energy (300 rpm)^11^ and interrupted high-energy (400 rpm, 30 min BM alternated with 10 min rest steps)^13^ configurations. Powders were processed with 5–20 stainless steel balls (*Ø*5–20 mm) and cylindrical vials (80 ml) at various ball-to-powder mass ratios (10 : 1–21.8 : 1), in nitrogen or argon inert atmosphere. Electrical power to BM motor was measured by a current ammeter to determine total energy consumption during the transient upon process startup,^19^ and the thermal energy stored in the BM container and contents was measured by a digital bomb calorimeter (Apex) at the end of ball milling, in order to calibrate the process friction and plasticity efficiencies for the model.^18^

**Fig. 2:**
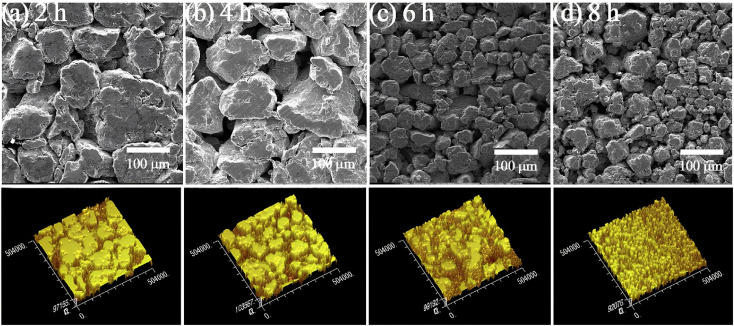
Micrographs of Al–Ni (1 : 3) particles after (a) 2 h, (b) 4 h, (c) 6 h and (d) 8 h of BM at 300 rpm^11^ (Top row): SEM secondary electron (SE) images; (Bottom row): Laser profilometer scans.

The size and shape of the particles was analyzed by removal of small samples (∼0.5 g) from the BM load upon brief process intervals in an inert gas (nitrogen) filled glove box to avoid potential contamination and pyrophoricity effects. These samples were imaged in 2D micrographs by scanning electron microscopy (SEM, Tescan VEGA) with secondary electrons (SE) and back-scattered electrons (BSE), as well as laser profilometry (Ambios, Fig. 2).^11^ Pairs of images of the respective samples were processed *via* grayscale edge detection for blob segmentation and particle identification methods, along with quadtree raster techniques for spatial occupancy measurement of the perimeter length *L* and section area *A* of each particle, *via* commercial scale-independent software.^20,21^ These blob features were used for particle classification into species according to their size and aspect ratio as in the next Section, and their population probability density function (pdf) characteristics were identified by standard statistical sampling and fitting algorithms.

### 2.2 Computational framework

**Fig. 3:**
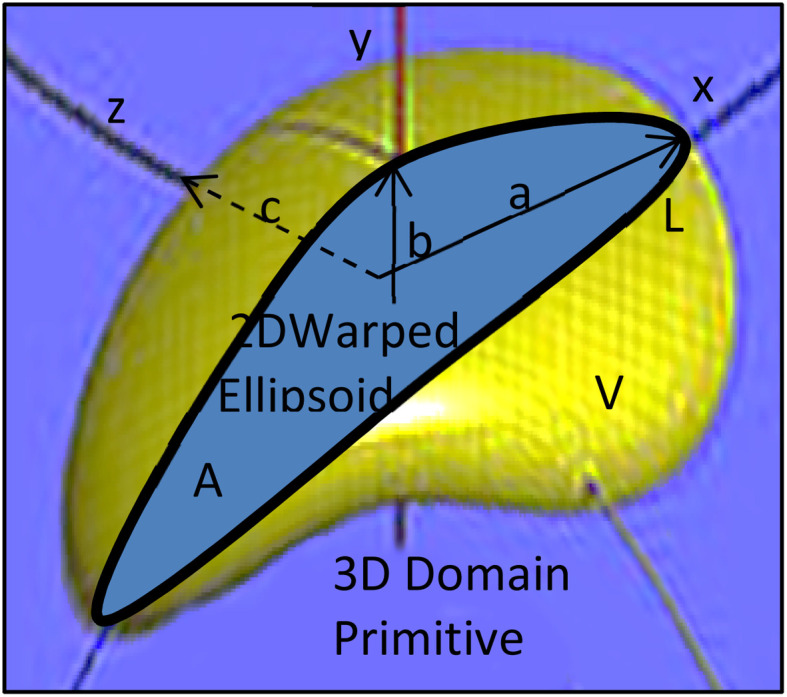
Warped ellipsoid domain primitive.

**Fig. 4:**
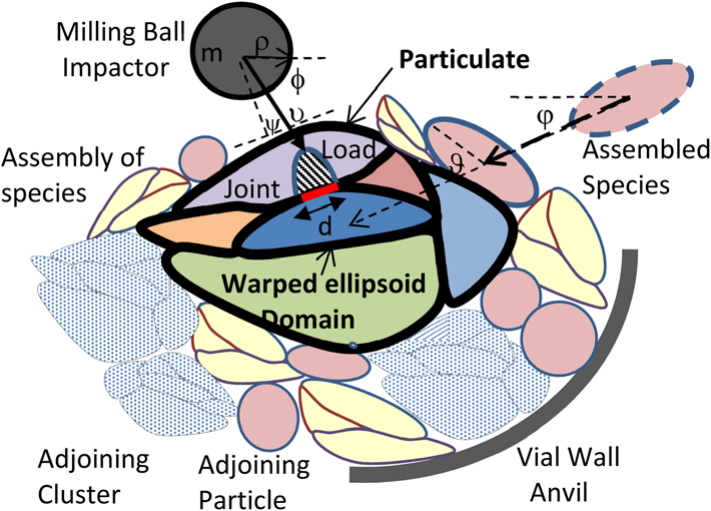
Particulate with assembly of adjoining particles, impactor and anvil (not to scale).

The predictive real-time model^18^ enables dynamic computational simulation of a representative particulate internal microstructure on a 2D section, matching the fractal structure of the planar laboratory micrographs above as it evolves during BM. The model represents monometallic domains originating from the initial powders in the BM vial by warped ellipsoids (Fig. 3), *i.e.* flexible primitives matching the evolving domain shapes from spheroidal powders to planarized lamellae, through parametric variation of their major axis lengths, angles and curvatures. However, despite this domain representation by 2D ellipsoids (*e.g.* of half-axis lengths *a* and *b*, Fig. 3), all mechanics and energetics computations are carried out on 3D solid topologies, assuming a third normal half-axis of length equal to the geometric mean of the others (*i.e.*
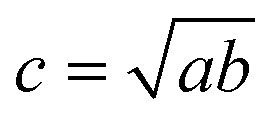
). Random assemblies of such domains are impacted by the milling balls and vial walls during BM (Fig. 4), according to impactor kinematics following experimentally assessed Maxwell–Boltzmann velocity pdfs.^19^ Such collisions create initial elastic Hertzian conditions at contacting surfaces of domains upon their compression, along with Coulomb friction and boundary slip. In the domain bulk, stored elastic energy determined by Castigliano strain methods is recast upon yield into plastic deformation work of work-hardening materials, leaving a balance of residual stress fields and kinetic energy restored to the BM impactors.

Friction and plasticity of domains dissipate mechanical energy as heat, raising local temperatures and altering material properties. When critical thresholds of dissipated specific energy per surface area are exceeded for monometallic or bimetallic contacts, micro-welded joints develop at domain boundaries, resulting in their integration and growth of the particulate. Upon subsequent collisions, when elastic tensile or shear stress fields developed at the joints exceed the energy thresholds, the micro-welds fracture separating the particulate into partial clusters. Thus, starting with the initial powders, these processes generate a diversity of particle species *i* (powders, clusters, particulates), each numbering *n*_*i*_ members for a varying total particle population in the vial 
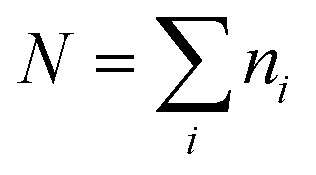
 at each time. The population fraction of each species is therefore *p*(*i*) = *n*_*i*_/*N* with 
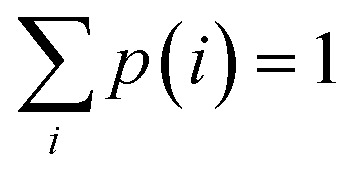
, reflecting the demographics of the BM contents. For quantitative further detail on this structural simulation, the interested reader is referred to its original account.^18^ The discussion below elaborates on certain of its stochastic aspects pertinent to the foundation of the size and shape model in the next Section.

### 2.3 Probabilistic structural modeling

#### 2.3.1. Impactor collision

**Fig. 5:**
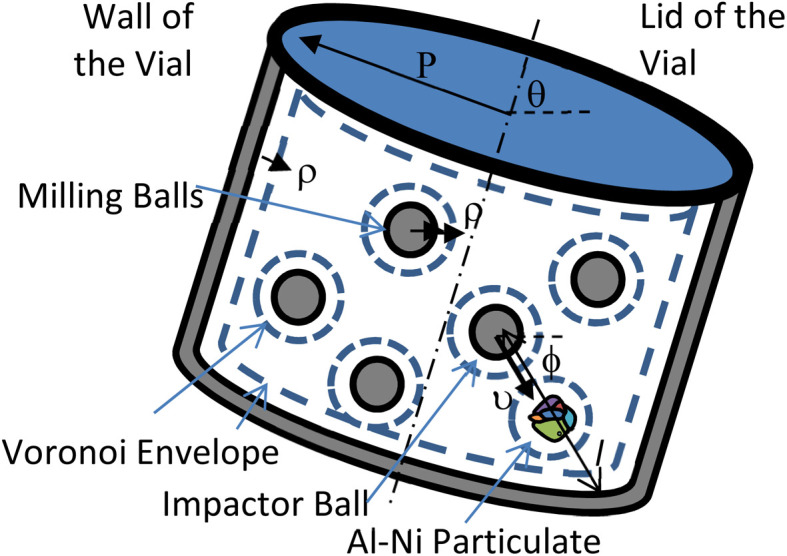
Collison schematic arrangement (not to scale).

The computational simulation above follows a Lagrangian frame fixed to the representative particulate examined, and uses Voronoi concepts^22^ for stochastic localization of the impactor balls and vial. The 2D position (*x*,*y*) and orientation *θ* of the particle inside the vial (or reversely the vial with respect to the particle, Fig. 5) is first randomly chosen. Next the balls are successively localized, by selecting the center position of each ball in the free space left by all envelope volumes, *i.e.* that of the particle and those inside the vial and around previously located balls. Envelope spaces are obtained by expanding the respective objects by one ball radius *ρ*, while shrinking the localized ball to the point of its center. Finally, the impactor motion orientation *ϕ* is randomly chosen (0.2π), and its projection from the particle location intercepts and defines the impactor ball as well as the anvil, which may be another ball or the vial cylindrical or lid wall (Fig. 5). The probability for each object to participate in a collision as impactor or anvil is thus proportional to the azimuthal angle it subtends to the particle position. The curvatures of the impactor and the anvil (1/*ρ* for balls, 1/*ρ* for cylindrical vial and 0 for flat lid walls) are essential for their Hertzian contact with the particle as mentioned previously. Finally, the average inter-collision period *t* for one impactor ball is defined by its flight time over its distance *l* to the anvil (Fig. 5), at a RMS velocity *v* determined from the kinetic energy *U* of the impactor (*i.e. U* = ½*mv*^2^, with *m* its mass), as selected in the next Section. Therefore, the estimated overall collision frequency in the vial is *f* = *Zυ*/*l*, where *Z* is the number of impactor balls in the BM vial.

#### 2.3.2. Particle assembly

Before each collision, the space around the particulate between the impactor and anvil is populated with randomly selected species members *i*, *i.e.* particles from the original powders or clusters generated in previous collisions, as per their current probability distribution *p*(*i*). Each chosen particle is attracted to the particulate at a random direction *φ* (0.2π) until it intercepts the assembly adjoining to the particulate (Fig. 4). Its orientation *ϑ* on the plane is adjusted to minimize the distance of its gravity center from that of the particulate, ensuring a stable contact to previous adjoining elements. The adjoining assembly is grown by further deposition of particles until the anvil or impactor surface are contacted. At this point the collision loads are applied to the assembly (Fig. 4) and the thermomechanical impact conditions causing surface friction and bulk deformation are implemented as mentioned above.^18^

#### 2.3.3. Species demographics

After each collision, joining and/or fracture in the assembly generates new particulate species *i* in terms of internal structure, as well as external size and shape, recorded by the simulation. At that time their counts *n*_*i*_ and population fractions *p*(*i*), along with those of the particles in the assembly depending on whether they have been integrated or not to the particulate by the collision, are properly updated by the species demographic bookkeeping. In between successive collisions of the examined particulate assembly with its impactor, however, the other *Z* − 1 impactor balls in the vial also act in parallel, causing *Z* − 1 additional collisions producing further species transformations similar to those already analyzed. Therefore, the real-time model after each impactor collision also implements another *Z* − 1 species transformations and updates, according to the outcomes of randomly chosen previous collision events examined during the BM simulation history. Such population augmentation *via* transformation similarity among successive collision events is further exploited in the establishment of the dynamic species size and shape model below.

## 3. Stochastic modeling of particle size and shape

### 3.1 Particle size and aspect ratio

If only the particulate external size and shape are of interest rather than its internal structure, the previous full real-time model can be simplified in its inter-domain transformation aspects towards a more efficient stochastic model. The size *s*_*i*_ of any species *i* particle (powder, cluster or particulate) is hereafter defined from its planar area *A*_*i*_ (or solid volume *V*_*i*_) as:1
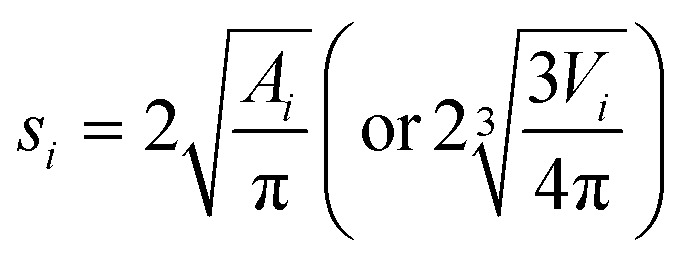


For a planar ellipsoid particle with half-axes *a*, *b* (Fig. 3) and *A* = π*ab*, its size is 
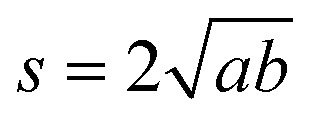
; this remains the same for a solid ellipsoid with third half-axis 
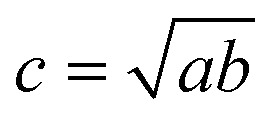
 and 
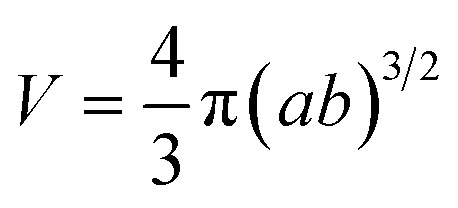
 as surmised above. This definition of size *s* makes it invariant under transformations of the species conserving its planar mass (*i.e.* area *A*) or density (*i.e.* volume *V*), which is assumed below for surface friction shear and bulk plastic deformation. For a species *i*, joining from or fracturing into *j* components of areas *A*_*j*_ (or solid volumes *V*_*j*_) yields, because of the assumed planar or solid mass conservation:

2 *A*_*i*_ = *A*_1_ + *A*_2_… + *A*_*j*_ ⇒ *s*_*i*_^2^ = *s*_1_^2^ + *s*_2_^2^… + *s*_*j*_^2^(*V*_*i*_ = *V*_1_ + *V*_2_… + *V*_j_ ⇒ *s*_i_^3^ = *s*_1_^3^ + *s*_2_^3^… + *s*_j_^3^

**Fig. 6:**
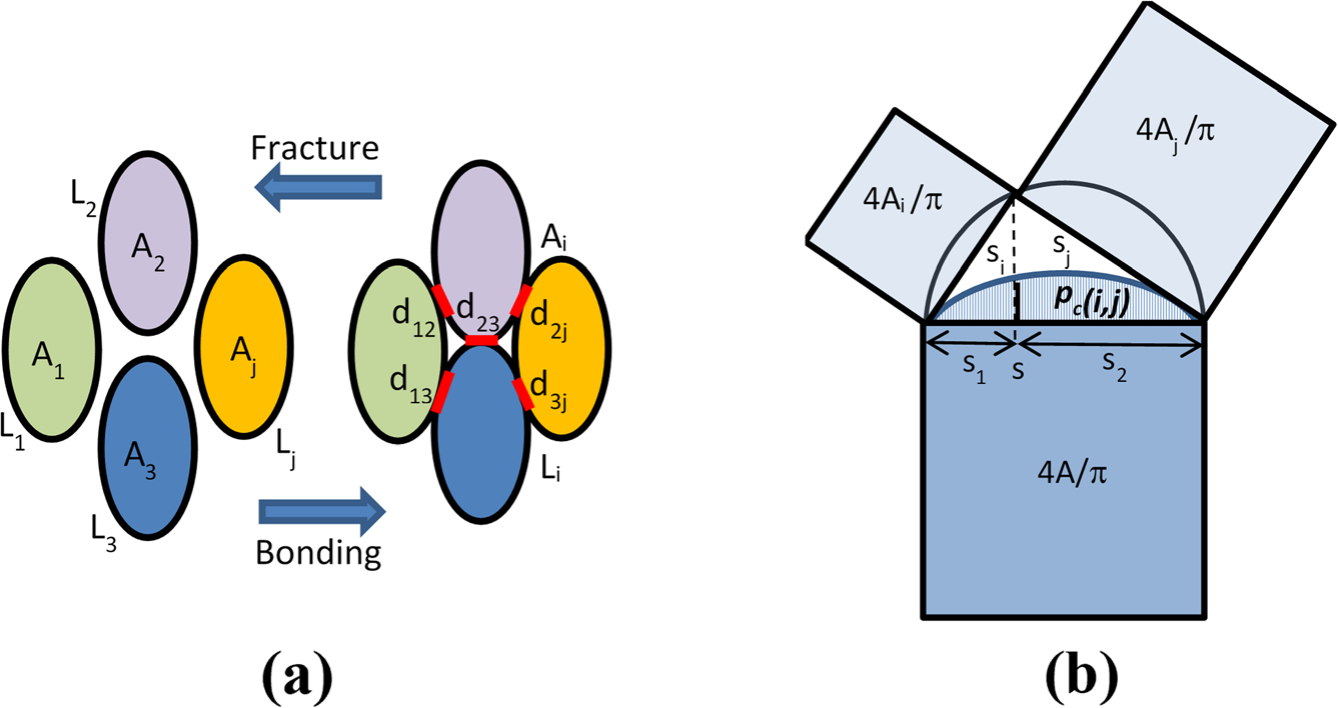
(a) Particle bonding and fracture. (b) Fracture size relationship and probability distribution.

*i.e.* component sizes add up in the power law (Pythagorean) sense^23^ (Fig. 6).

The shape of species *i* is defined through its aspect ratio *r*_*i*_, from its planar perimeter *L*_*i*_ (or solid surface *S*_*i*_) and size *s*_*i*_ as follows:3
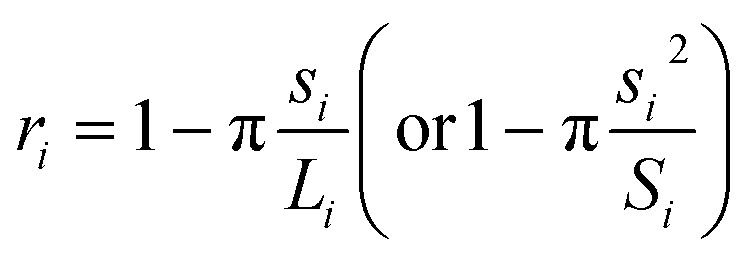


It can be shown that always *r* ≥ 0, ranging between *r* = 0 for a circular or spherical species, and *r* = 1 for an ideal fractal species boundary (*i.e.* with Hausdorff dimension >1 for a planar curve, or >2 for a solid surface^23^) where *L* (or S) → ∞. For a planar ellipsoid with *L* = π(*a* + *b*), the aspect ratio is 
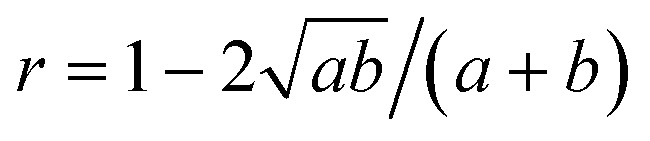
. For a species *i* assembled from or separated into *j* components of sizes *s*_*j*_ and perimeters *L*_*j*_ (or surfaces *S*_*j*_) through *k* joints of length *d*_*k*_ (or areas *D*_*k*_), this yields (Fig. 6):4
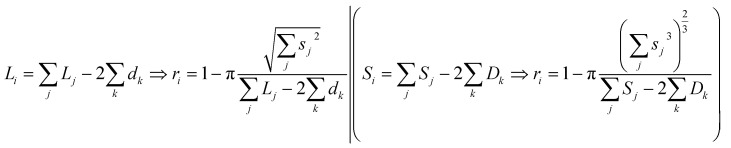


### 3.2 Ball milling energetics and assumptions

Thus, modeling of the BM process is implemented *via* successive stochastic transformation operations on an orthogonal raster of species *i*(*s*_*i*_,*r*_*i*_) with population fraction *p*(*i*) = *p*(*s*_*i*_,*r*_*i*_), initially defined by the particle statistics of the original powders in the BM vial. The dynamics of size and aspect ratio are simulated using their 2D definitions above, congruent with the planar laboratory micrographs (Fig. 1 and 2) and real-time structural model predictions. Thermomechanical steady state conditions are assumed during the BM period, and variation of composition, material properties and process conditions during transients are ignored. Energy for species transformations is provided by the milling ball and vial motion, while enthalpic effects from residual elastic fields, phase change, chemical reaction, thermoelectricity *etc.* are neglected. The impactor kinematics are modeled by an experimentally validated Brownian-like probability density function for kinetic energy *U*:^19^5
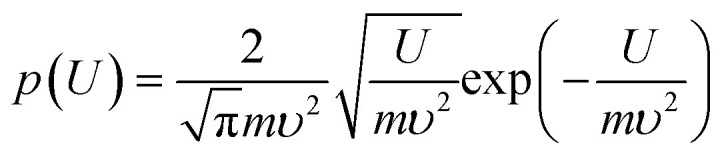
where *m* is the impactor mass and *υ* the RMS velocity, determined and calibrated from power measurements on the BM device during the starting transient as mentioned before.^19^ The collision incidence angle *ψ* of the impactor normal to the particle surface (Fig. 4) is selected from a uniform pdf in the range (−π/2, π/2).

The stochastic model operates upon sequential discrete collision events, steadily timed at a period 1/*f* = *l*/*Zυ* as in the previous Section. For each collision, a pair of values for the impactor kinetic energy *U*_in_ and *U*_out_ before and after impact respectively are randomly chosen *via* the pdf of eqn (5), with *U*_in_ > *U*_out_, along with an incidence angle *ψ*. The specific mechanical work of impact per planar area of processed particles is computed over their average area *A* from their RMS size *s* according to their current population pdf *p*(*s*_*i*_):6
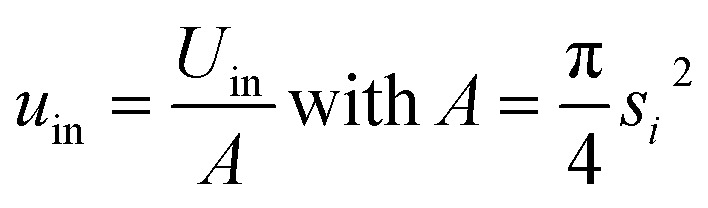


The energy difference Δ*U* = *U*_in_ − *U*_out_ is to be dissipated as heat during the collision event through friction slip and plastic deformation, occasionally causing particle bonding or fracture and generating new species as in the following paragraphs. For this purpose, a first species object *i* is randomly selected for each collision from the current population demographics raster *p*(*s*_*i*_,*r*_*i*_), on which the size and shape transformations below are implemented.

### 3.3 Friction and plasticity effects

Surface friction at the impactor and/or anvil contacts with the particle object *i*, as well as at the interfaces among its internal domains, cause thermal loss of an energy amount *ΔU*_f_:^18^7
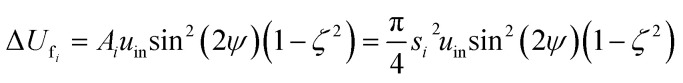
where the tribologic factor *ζ* depends on surface friction coefficients and compression/shear stress ratios at the interfaces, and is in the range (0, 1) for energy-dissipating surface slip to occur. Similarly, bulk plastic deformation and inelastic hysteresis of the particle *i* internal domains upon impact generate a heat amount Δ*U*_p_:^18^8
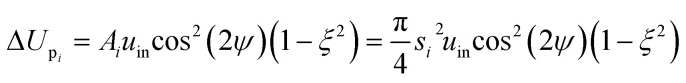
where the plasticity factor *ξ* depends on the elastic and strain-hardening moduli and the yield points of the domain materials, and is also within (0, 1) for energy loss by plastic yield and deformation.

**Fig. 7:**
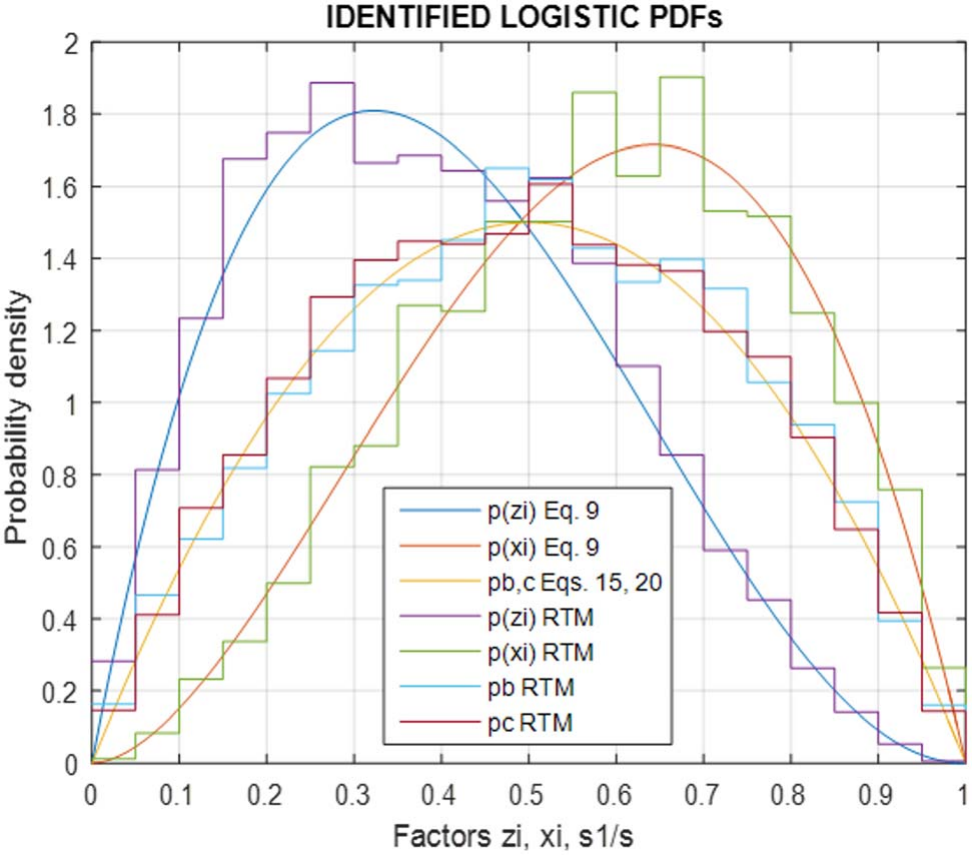
Probability density functions of tribological factor *ζ*, plasticity factor *ξ* and fracture size *s*_1_/*s* resulting from real-time model and fitted by eqn (9), (15) and (20).

Both factors *ζ* and *ξ* are dependent on the internal structure and composition, as well as material properties and loading conditions of the particle species, which are explicitly considered by the real-time simulation,^18^ but not by the present stochastic model. Therefore, their values are randomly selected from exponential logistic function pdfs (Fig. 7), statistically matched to the respective results of the real-time model above, while simulating the same BM process examined:

9 *p*(*ζ*) = (*α* + 1)(*α* + 2)*ζ*(1 − *ζ*)^*α*^, *p*(*ξ*) = (*β* + 1)(*β* + 2)*ξ*^*β*^(1 − *ξ*)

where the exponents *α* and *β* are calibrated *via* computational runs as in the next Section. Last for species *i* the available energy is reduced by the dissipated heat Δ*U*_d_ of eqn (7) and (8) and updated as:

10 Δ*U* ← Δ*U* − ΔU_d_*i*__ where Δ*U*_d_*i*__ = Δ*U*_f_*i*__ + Δ*U*_p_*i*__

**Fig. 8:**
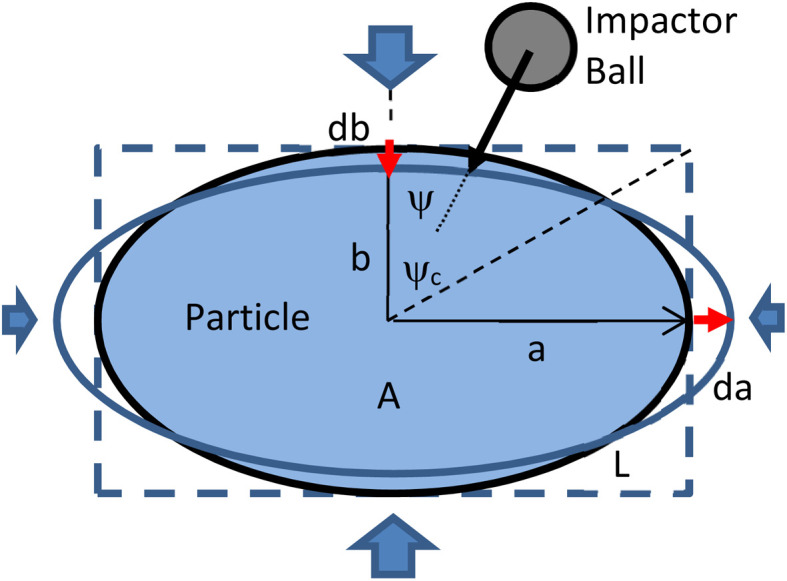
Schematic arrangement for plastic deformation (not to scale). (a and b) Half-axes of ellipsoidal particle d*a*, d*b*: elongation and compression deformation of half axes *Ψ*, *Ψ*c: incidence angle and critical incidence angle *A*: particle area.

Surface friction shear is assumed not to alter the particle area and perimeter length, and therefore preserves its size and aspect ratio. However bulk plastic deformation, while maintaining the density and thus the particle area *A*_*i*_ and size *s*_*i*_, does alter its perimeter *L*_*i*_ and shape *r*_*i*_. For an ellipsoid of fixed area *A*_*i*_ (Fig. 8), strain along its half-axis lengths *a*, *b* upon deformation yields:11
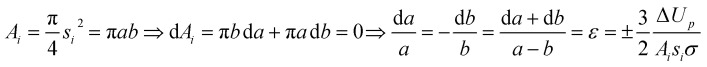
where *ε* and *σ* are an equivalent strain and yield stress of the particle. Strain of the perimeter *L*_*i*_ is:12
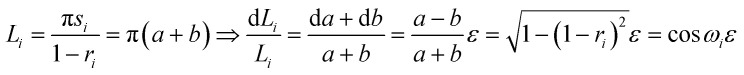
with *ω*_*i*_ ≡ sin^−1^(1 − *r*_*i*_). Therefore, the change in the aspect ratio *Δr*_*i*_ of the particle upon plastic deformation is determined as:13
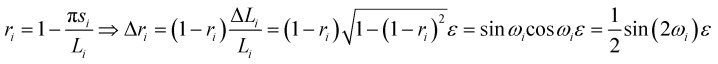


The sign of deformation *ε* (extension or compression) of the major axes in eqn (11)–(13) depends on the incidence angle *ψ* of the impact load (Fig. 8) exceeding a critical value:14
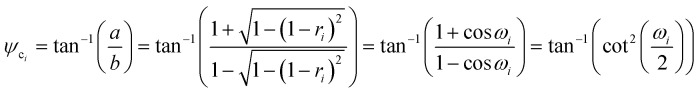


*i.e.*, *ε* > 0 for *ψ* in (−*ψ*_c_, *ψ*_c_) and *ε* < 0 for *ψ* in (−π/2, −*ψ*_c_) or in (*ψ*_c_, π/2). It is noteworthy here that for uniformly random incidence *ψ* collisions and oblate objects (*r*_*i*_ > 0, *e.g. a* > *b* in Fig. 8), *ψ*_c_ > π/4 and the first angle range is wider than the second. This condition yields a higher probability for further planarization of such particles (*i.e.*, Δ*r*_*i*_ > 0), with a lower probability for their spheronization (Δ*r*_*i*_ < 0), thus inducing quicker planarization for more oblate objects (higher *r*). This theoretical result is in agreement with and explains statistical data from prior computer simulations.^17^

### 3.4 Bonding and fracture dynamics

Bonding transformations are applied to particle pairs *i* and *j* joined into a single particle, while multi-particle bonding is implemented by sequentially joining of the outcome to a third particle *k etc.* Thus, along with the already selected particle *i*, a second particle *j* is randomly chosen from the current population raster according to probability *p*(*j*) = *p*(*s*_*j*_,*r*_*j*_). To be bonded, the two particles need to be located with touching boundaries. The probability *p*_b_ for such bonding of particles *i* and *j* is proportional to the perimeter length of each particle with a normalizing contact factor *γ*, and thus follows a logistic parabola function of their respective sizes (Fig. 7), *i.e.*:15
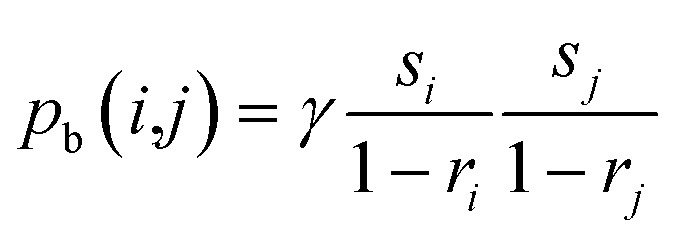


Once in touch and upon collision of the impactor, initial elastic impact loading of the particles creates compressive linear boundary contact along their interface of length *d*_*ij*_ (Fig. 4 and 6), given by Hertzian theory.^18^ When in addition the dissipated specific energy per contact length *Δu*_d_ exceeds a critical value for bonding, then a micro-welded joint forms along the contacting boundaries:16
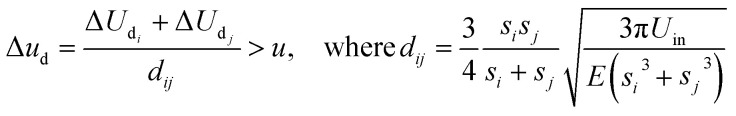
where *E* is an equivalent elastic modulus and *u* the specific bonding energy of the particle pair materials. The probability for this joint energy threshold to be exceeded can be represented by a logistic sigmoid distribution, which is simplified to a saturation condition as:

17 *p*_u_(Δ*u*_d_) = [1 + e^−*k*(Δ*u*_d_−*u*)^]^−1^(≈0 for Δ*u*_d_ < *u*, 0.5 for Δ*u*_d_ = *u*, 1 for Δ*u*_d_ > *u*)

because of the large exponent factor *k*. Therefore the composite probability for a successful joint between particles *i* and *j* is *Δp*_b_ = *p*(*s*_*i*_,*r*_*i*_) × *p*(*s*_*j*_,*r*_*j*_) × *p*_b_(*i*,*j*) × *p*_u_(Δ*u*_d_) as determined above.

The size *s* and aspect ratio *r* of the bonded new particle species are according to eqn (2) and (4) (Fig. 6):18
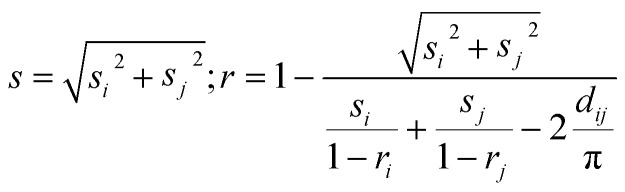


Therefore the probability for a new species (*s*,*r*) to be generated out of all possible combinations of bonded particles *i* and *j* with suitable sizes and shapes as above is:19
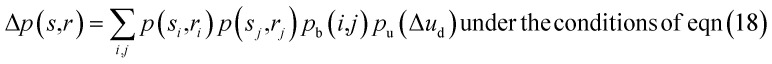


Fracture transformations of particles by abrasion, compression and impact fatigue^1,2^ also take place by their separation into two new species *i* and *j* at a time, while splitting into multiple particles is implemented *via* subsequent dichotomies of each species. The probability for cracking of a particle (*s*,*r*) along its previous surface boundary joints and/or its internal domain thickness is proportional to the perimeter length and size of the species, with a normalizing parting factor *δ*. Moreover, the probability *p*_c_ for such a fracture to generate two species (*s*_*i*_,*r*_*i*_) and (*s*_*j*_,*r*_*j*_) conforming to the previous size and aspect ratio constraints (eqn (18) and Fig. 6) follows a logistic function of the areas *A*_*i*_, *A*_*j*_ of the two parts (Fig. 6b), such that:20
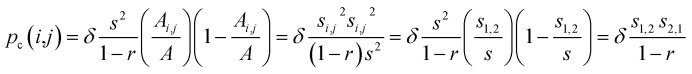
where *s*_*i*,*j*_ = *s*_*i*_ or *s*_*j*_, and *s*_1,2_ = *s*_1_ or *s*_2_ are their orthogonal projections on *s* (Fig. 6b). Last, the model also assumes that the dissipated energy *Δu*_d_ in the fracturing particle (eqn (10)) must exceed the barrier *u* (eqn (16)) for joint separation, with the respective probability given by eqn (17). Thus the composite probability for a particle (*s*,*r*) to fracture into parts *i* and *j* is *Δp*_c_ = *p*(*s*,*r*) × *p*_c_(*i*,*j*) × *p*_u_(Δ*u*_d_). The size and aspect ratios of these parts conform to the previous relationships (Fig. 6 and eqn (18)).

Therefore upon each collision, the stochastic model performs the species transformations above, updates the population demographics raster *p*(*s*,*r*), and reduces the available energy Δ*U* accordingly (eqn (10)). While Δ*U* > 0 the calculation repeats more species transformation steps as in paragraphs 3.3 and 3.4, until Δ*U* is depleted. At this time the model proceeds with the next collision by repeating the energetic computations from paragraph 3.2, until the conclusion of the BM processing period.

## 4. Results and discussion

### 4.1 Model calibration and ball milling tests

As described in Section 2, BM experimentation was employed to test the real-time structural model and the statistical size and shape formulation of the particles for a trial case of Al–Ni powder processing at 1 : 1 molar ratio and at 300 rpm. The laboratory measurements of transient power and thermal steady state were initially used to calibrate the real-time model effective friction and plastic yield parameters mentioned previously,^18^ and the structural simulation was run to emulate the BM process computationally. The resulting statistics for the pdfs *p*(*ζ*) of the tribological factor *ζ* and *p*(*ξ*) of the plasticity factor *ξ* (eqn (9)), as well as the bonding *p*_b_(*i*,*j*) (eqn (15)) and fracture *p*_c_(*i*,*j*) (eqn (20)) occurrence pdfs between species *i* and *j*, which are inaccessible experimentally in real time, were recorded during simulation. These were used next to calibrate the values of the friction *α* = 2.1 and plasticity *β* = 1.8 exponents, along with the contact *γ* = 1.82 10^−4^ μm^2^ and parting *δ* = 8.1 10^−4^ μm^−2^ factors, by fitting the respective logistic functions of the stochastic model to the statistical data of the simulation (Fig. 7). The physical parameters of the BM materials, *i.e.* mass *m* = 4.14 g, elastic modulus *E* = 120 GPa, yield stress *σ* = 78.7 MPa and bonding/fracture threshold *u* = 6.4 10^−6^ J μm^−1^ were calculated *via* the molar law of mixtures.^18^

**Fig. 9:**
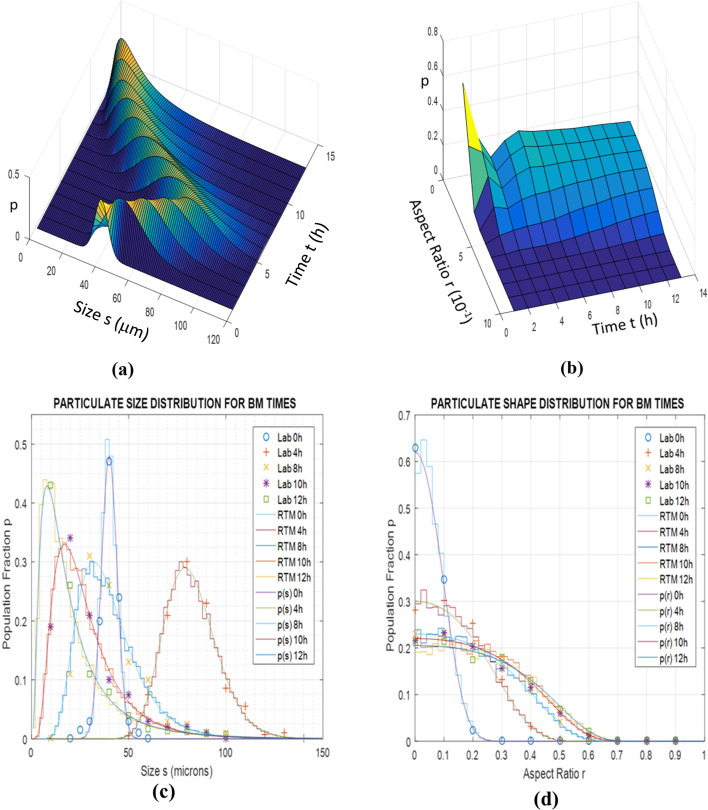
Population fraction pdfs *p* for calibration BM tests (Al–Ni 1 : 1, 300 rpm) (a) stochastic model results for *p* as function of size *s* and time *t* b. Stochastic model results for *p* as function of aspect ratio *r* and time *t* (c) laboratory data, RTM simulations and stochastic results of size *s* for various times *t* (d) laboratory data, RTM simulations and stochastic results of aspect ratio *r* for various times *t.*

The stochastic model results for this trial case are comprehensively illustrated in Fig. 9, in terms of the species population fraction pdf *p* distribution for the range of BM times *t* and particle sizes *s* (Fig. 9a) or aspect ratios *r* (Fig. 9b). Fig. 9c and d compare the distribution *p* of particle size *s* and aspect ratio *r* respectively for the laboratory measurements, real-time model simulations and stochastic model results, at various BM time sections *t* (0, 4, 8, 10 and 12 h). Classification of particle species in the experimental micrographs is performed on an orthogonal size-shape raster of elements measuring Δ*s* = 10 μm, Δ*r* = 0.1, while for the real-time model the tessellation resolution is Δ*s* = 2 μm, Δ*r* = 0.02 and for the stochastic formulation is Δ*s* = 1 μm, Δ*r* = 0.01. Results are recorded every Δ*t* = 1 h of BM time over a range of several hours. Execution of the structural simulation on standard desktop computer hardware keeps up with laboratory BM rates up to ∼10 h, after which it falls behind process speeds due to increasing complexity of the representative particulate structure. The stochastic model runs steadily faster than actual BM processing by a factor of ∼8–15 depending on the size range. Matching of the experimental measurements by the simulation predictions and the size and shape estimates confirms calibration of the real-time model and the stochastic formulation for the specific BM processing conditions.

### 4.2 Validation and results analysis

**Fig. 10:**
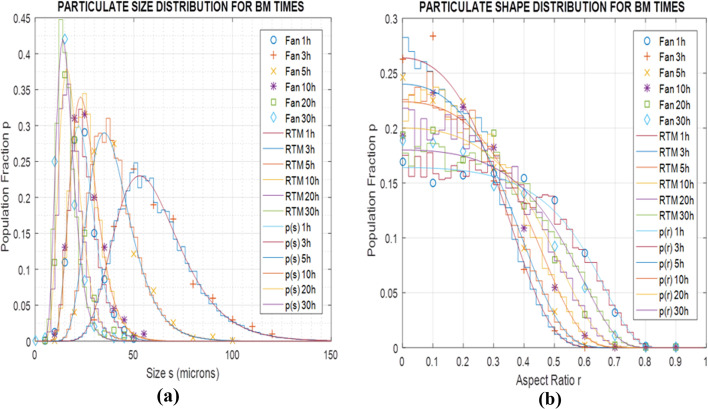
Population fraction pdfs *p* for validation BM tests using Fan *et al.*^13^ (Al–Ni 1 : 1, 400 rpm) (a) literature data, RTM simulations and stochastic results of size *s* for various times *t* (b) literature data, RTM simulations and stochastic results of aspect ratio *r* for various times *t*.

**Fig. 11:**
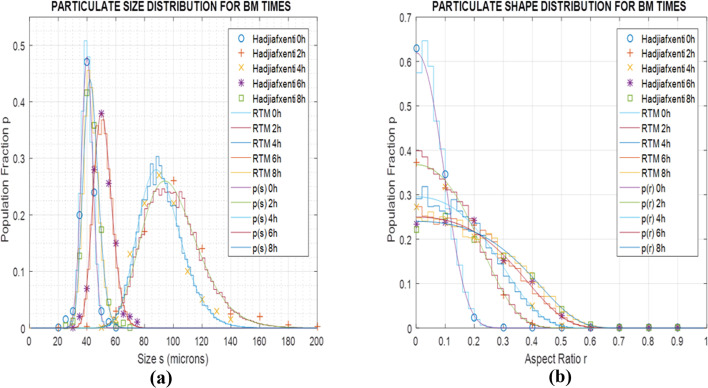
Population fraction pdfs *p* for BM tests using Hadjiafxenti *et al.*^11^ (Al–Ni 1 : 3, 300 rpm) (a) literature data, RTM simulations and stochastic results of size *s* for various times *t* (b) literature data, RTM simulations and stochastic results of aspect ratio *r* for various times *t*.

Validation of the size and shape predictions of both models was subsequently carried out using literature data for BM at different revolution speeds and powder molar ratios. In these comparisons the structural model parameters were adjusted through a simple testing procedure,^18^ while the logistic exponents and factors of the stochastic model were fitted to the computational results. For BM at 400 rpm and Al–Ni molar ratio maintained at 1 : 1, for which particle SEM micrographs are reported (Fan *et al.*^13^), Fig. 10a and b illustrate the population fraction profiles *p* of size *s* and shape *r* of experimental data, real-time simulations and stochastic model predictions with parameter values *α* = 2.17, *β* = 1.72, *γ* = 3.1 10^−4^ μm^2^ and *δ* = 10.2 10^−4^ μm^−2^. Similarly, for BM speed maintained at 300 rpm and Al–Ni powder ratio changed to 1 : 3 (Hadjiafxenti *et al.*,^11^Fig. 2), Fig. 11a and b show the demographics of size and shape of micrograph measurements, real-time and stochastic model estimates with *α* = 2.05, *β* = 1.86, *γ* = 1.2 10^−4^ μm^2^ and *δ* = 6.3 10^−4^ μm^−2^. In both cases, comparison of the experimental with computational results attests to the validity of the two models, with some parameter adjustment of statistical factors needed for the stochastic formulation.

**Fig. 12:**
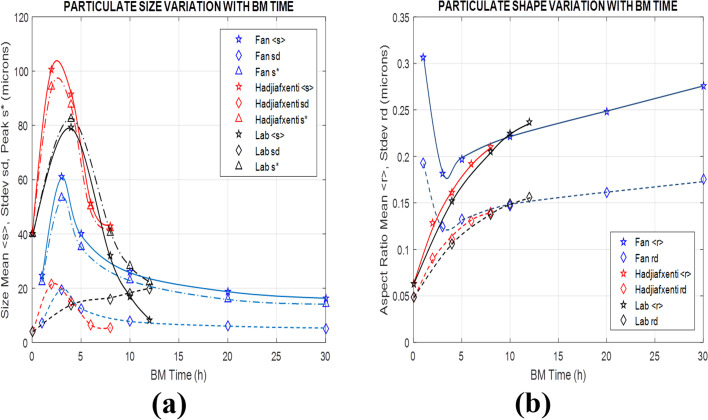
Time variation of particle size and shape in Fan,^13^ Hadjiafxenti^11^ and lab BM tests (a) mean *s*, standard deviation *s*_d_ and most probable value s* of size *s* (b) mean *r* and standard deviation *r*_d_ of aspect ratio *r*.

Finally, for all three BM test conditions above *i.e.* Fan's^13^ (Fig. 10), Hadjiafxenti^11^ (Fig. 11) and laboratory (Fig. 9) results, Fig. 12a and b summarize the time variation of the statistical metrics for particle size and shape, *i.e.* the mean value, standard deviation and most probable size and aspect ratio, as derived by the validated stochastic model. As it appears in Fig. 9c, 10a and 11a, in these BM tests and at a certain time snapshot the particle size distribution exhibits a log-normal shaped profile, consistent with previous observations,^4^ and starting with a Gaussian-like initial pdf of the powders (Figs. 9c and 11a).^16^ Similarly in Figs. 9d, 10b and 11b, the respective aspect ratio demonstrates a hyperbolic-normal distribution, with highest likelihood for spheroidal shaped particles (most probable r* → 0).^17^

After the beginning of the process and in the first few hours, the size profiles *p*(*s*) initially broaden drastically, with their mean value, standard deviation and most probable *s* all increasing rapidly with time (Fig. 12a), confirming previous arguments.^2^ This is due to the growing friction and plastic deformation effects, raising the BM temperature and softening the particle materials, with bonding rates prevailing over fracture events, thus generating larger and more intricately shaped particles. This similarly leads the aspect ratio distributions *p*(*r*) to initially spread out quickly (Fig. 12b), with the exception of high-energy BM,^13^ where the original rougher powder particles tend to coagulate into spheronized bonded particulate agglomerates at the early process stages.^17^ As time progresses, successive impactor collisions lead to work-hardening of the BM materials, in agreement with prior accounts,^2^ along with formation of bimetallic solid solutions by progressing diffusion at interface contacts and joints, causing growing fracture rates to gradually balance bonding events. This results in progressive narrowing of the size pdfs *p*(*s*) in Fig. 9c, 10a, 11a over longer BM periods, with their statistical measures *s*, *s*_d_ and s* generally decreasing with time (Fig. 12a) in accordance with previous results.^15,16^ The exception here is in standard deviation of size at the calibration tests, increasing apparently because of a persistent population of larger welded and hardened particles (Fig. 9c), similar to ref. 4. Oppositely to size effects, fracture of cracking particulates creates rough-shaped particles with their characteristics *r* and *r*_d_ increasing slowly (Fig. 12b), reflecting gradual broadening of the shape *p*(*r*) profiles in Figs. 9d, 10b and 11b.

As for varying BM process conditions, an increase of the low-energy rotation speed from 300 to 400 rpm high-energy BM,^13^ appears to significantly enhance the friction and plastic deformation effects, along with respective parameters in calibration of the real-time model. In the stochastic formulation this is reflected in slightly varied exponents *α* and *β*, both shifting the weight of logistic distributions in Fig. 7 to lower tribological and plasticity factors *ζ* and *ξ* (eqn (9)), thus increasing heat dissipation (eqn (7) and (8)), as previously observed.^15,16^ At the same time drastically higher joining contact *γ* and larger parting *δ* factors indicate higher bonding and fracture rates with increased revolution speed. On the contrary, a modified Al–Ni ratio from 1 : 1 to 1 : 3,^11^*i.e.*, less Al and more Ni content, reduces friction slip and plastic yield effects, changing logistic exponents *α* and *β* in the opposite direction, *i.e.*, towards lower heat dissipation. Finally, the reduced bonding rates due to the lower Al content, and fracture proclivity because of higher Ni concentration, is also reflected in the diminishing respective *γ* and *δ* factors in the stochastic model.

### 4.3 Comparative evaluation

Therefore, the previous account has introduced two computational modeling tools for studying bimetallic particle species demographics, their population evolution and dependence on the BM process conditions: the first model is a full structural simulation of the internal domain microstructure in a representative particulate, with probabilistic representations of impactor collisions, particle assembly and amplification of species; the second model is a key improvement over the numerical^18,19^ and experimental literature^40–42^ as it provides a simpler stochastic formulation of particle external size and shape only, with impactor energetics, friction and plasticity effects. along with bonding and fracture transformations of species, rendered by proper probability distribution functions. The full simulation is multi-physics based, enables prediction of both internal and external particulate structure, and is experimentally calibrated *via* two physically meaningful parameters. However, it yields more noisy estimates of particle size and shape, and is more computationally demanding, maintaining real-time execution capability only up to ∼10 BM hours. The stochastic model, on the other hand, has higher computation efficiency and yields smoother profiles of particle populations, running 8–15 times faster compared to the BM numerical simulation or experiments, and therefore enabling a true real-time in-process model. However, it is based on statistical formulations of the BM phenomena, and relies on calibration of four logistical parameters on the basis of experimentally unmeasurable transformation rate data, provided by and thus requiring access to the full simulation model.

## 5. Conclusion and applications

In summary, two complementary models were implemented, calibrated and validated in low- and high-energy BM of Al–Ni powders at different molar ratios, through a laboratory setting *via* SEM micrograph image processing, along with related bibliographic data. Their results offer insights to the role of friction, plastic deformation as well as joining and cracking mechanisms, in the particle size and shape distributions, their dynamics and reliance on material and process parameters. Since such attributes are of importance to operational performance of particle products, but are also difficult or impossible to observe non-invasively and non-destructively in-process during BM, the highlighted predictive stochastic model offers new off-line material design and optimization capabilities for a variety of applications.

Future research should explore integrating machine learning algorithms with the stochastic framework to enhance predictive accuracy across diverse material systems and processing conditions. Additionally, experimental validation through closed-loop ball milling trials – using the model's real-time predictions for adaptive process control – could decisively bridge the gap between computational modeling and industrial-scale implementation.

At the same time and given its real-time implementation potential, the model provides in-process observer tools for surrogate feedback control of particle size and shape during BM fabrication.

## Conflicts of interest

There are no conflicts to declare.

## Data Availability

The data supporting this article have been included in the main manuscript.
